# The complete chloroplast genome of *Quercus fabri* (Fagaceae) from China

**DOI:** 10.1080/23802359.2019.1660921

**Published:** 2019-09-06

**Authors:** Yang Xu, Hao Chen, Min Qi, Wei Su, Yue Zhang, Fang K. Du

**Affiliations:** College of Forestry, Beijing Forestry University, Beijing, PR China

**Keywords:** Oak, chloroplast genome, next-generation sequencing

## Abstract

The complete chloroplast (cp) genome sequence of *Quercus fabri* Hance has been characterized in this study. The length of cp genome was 161,227 bp, containing a large single-copy region (LSC) of 90,539 bp and a small single-copy region (SSC) of 19,048 bp, which were separated by a pair of 25,820 bp inverted repeat regions (IRs). The genome contained 134 genes, including 86 protein-coding genes, 40 tRNA genes, and 8 rRNA genes. The overall GC content is 36.82%. Further, phylogenetic analysis suggested that *Q. fabri* is clustered to section *Quercus*.

As an oriental oak, *Quercus fabri* Hance primarily spreads over mixed mesophytic forests below altitudes of 100–1900 meters in South and Southwest China, according to specimen records of 14 provinces. Photophilous, drought resistant and strong budding make it a pioneer species during secondary succession, thus it plays an important role in the succession of the plant community. However, there is no complete chloroplast (cp) genome of *Q. fabri* in GenBank database. Here, we firstly reported the complete cp genome of *Q. fabri*.

Fresh leaves of *Q. fabri* were sampled from Sanjiangyan, Guizhou province, China (26.3547N, 107.5103E). Voucher specimens were deposited at the herbarium of Beijing Forestry University (SJYA15). The genomic DNA was extracted and then sequenced using Illumina-HiSeq 2000, with a 150 bp paired-end running (Du et al. 2015). NovoPlasty was used to assemble the cp genome (Dierckxsens et al. 2017), with the cp genome of *Quercus aliena* BI. Hayata as the reference (Genbank accession no. KP301144) (Lu et al. 2016). We annotated the assembled sequence with CPGAVAS (Liu et al. 2012). The annotated cp genome sequence has been submitted to NCBI with an accession number of MK922346. A circular map of the genome was generated with OGDRAW (Lohse et al. 2013).

The complete cp genome of *Q. fabri* is 161, 227 base pairs (bp) in length, containing a large single-copy region (LSC) of 90, 539 bp, a small single-copy region (SSC) of 19, 048 bp, and two inverted repeat regions (IRs) of 25,820 bp. The new sequence possesses 134 genes, including 86 protein-coding genes, 8 rRNA genes, and 40 tRNA genes. Among them, four rRNA genes (i.e. *16S*, *5S*, *4.5S*, and *23S* rRNA), seven protein-coding genes (i.e. *rpl2*, *rpl23*, *ycf2*, *ndhB*, *rps7*, *rps12* and *ycf1*), and seven tRNA genes (i.e. *trnI-CAU*, *trnL-CAA*, *trnV-GAC*, *trnI-GAU*, *trnA-UGC*, *trnR-ACG*, *trnN-GUU*) occur in double copies. The overall GC-content of the cp genome is 36.82%, while the corresponding values of the LSC, SSC, and IR regions are 34.66, 30.94, and 42.78%, respectively.

To further investigate its phylogenetic position, a maximum-parsimony tree was constructed based on complete cp genome sequences of 14 Fagaceae species using PAUP (Swofford 2003) with 1000 bootstrap replicates. Here, we aligned all 14 sequences using MAFFT (Katoh and Standley 2013). Our results showed that *Q. fabri* is clustered to section *Quercus* with 100% bootstrap support (Figure 1). Moreover, the phylogenetic positions of other species consist of a previous study by Denk et al. (2017).

**Figure 1.**
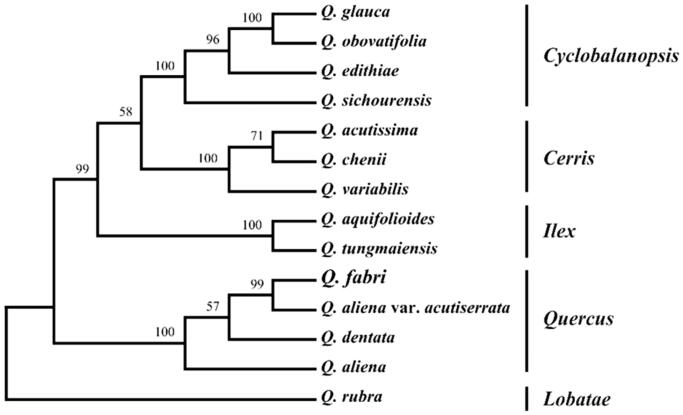
Phylogenetic relationships of 14 Fagaceae species based on chloroplast genome sequences. Bootstrap support is indicated for each branch. GenBank accession numbers: *Quercus glauca* (KX852399.1), *Quercus obovatifolia* (MG356785.1), *Quercus edithiae* (KU382355.1), *Quercus sichourensis* (MF787253.1), *Quercus acutissima* (MH607377.1), *Quercus chenii* (MF593894.1), *Quercus variabillis* (KU240009.1), *Quercus aquifolioides* (KP340971.1), *Quercus tungmaiensis* (MF593893.1), *Quercus aliena* var. *acutiserrata* (KU240008.1), *Quercus dentata* (MG967555.1), *Quercus aliena* (KP301144.1), and *Quercus rubra* (JX970937.1).

## Geolocation information

The samples in this study were from Sanjiangyan, Guizhou province, China (26.3547N, 107.5103E).
